# Intestinal tuberculosis with multiple intestinal perforations in a 5-month-old infant: The role of metagenomic next-generation sequencing: a case report and literature review

**DOI:** 10.1097/MD.0000000000051329

**Published:** 2026-11-06

**Authors:** Bei Wang, Yipaguli Simijiang, Gulibiya Kaheman, Yanfei Cui, Yusufu Akemu, Nuerai Shawutali

**Affiliations:** a Department of Critical Care Medicine, Pediatric Research Institute of Xinjiang Uygur Autonomous Region, Children’s Hospital of Xinjiang Uygur Autonomous Region, Xinjiang Hospital of Beijing Children’s Hospital, Urumqi, Xinjiang, China.

**Keywords:** infant, intestinal perforation, intestinal tuberculosis, malnutrition, metagenomic next-generation sequencing

## Abstract

**Rationale:**

Intestinal tuberculosis (ITB) is a chronic infectious disease caused by Mycobacterium tuberculosis complex and is relatively rare in infants. However, severe complications such as intestinal perforation can pose significant clinical challenges. We report the clinical features, diagnosis, and therapeutic management of intestinal perforation caused by ITB in an infant.

**Patient concerns:**

A 5-month-old male infant presented with fever and abdominal distension, which progressed to intestinal obstruction and multiple perforations involving the distal ileum and appendiceal region. The clinical presentation was atypical.

**Diagnoses:**

The diagnosis was established through surgical exploration and metagenomic next-generation sequencing (mNGS).Conventional investigations, including the tuberculin skin test using purified protein derivative and interferon-gamma release assay, were negative. mNGS of peritoneal drainage fluid detected mycobacterium tuberculosis complex with 2868,520 sequence reads, and Ziehl–Neelsen staining of mesenteric nodule specimens was positive for acid-fast bacilli.

**Interventions:**

The infant underwent surgical intervention for the intestinal perforations, followed by the standard four-drug anti-TB regimen (rifampicin, isoniazid, pyrazinamide, and ethambutol) together with linezolid, imipenem, and amikacin.

**Outcomes:**

The infant showed substantial clinical improvement, with weight gain and encouraging developmental progress; although the ileostomy remains in situ, closure is planned after full nutritional recovery. To our knowledge, this is among the youngest reported cases of postnatally acquired primary ITB presenting with multiple intestinal perforations in an infant without a known history of TB exposure.

**Lessons:**

In infants with severe unexplained intra-abdominal infection and inconclusive conventional TB investigations, mNGS may facilitate the early diagnosis of ITB. Timely surgical intervention combined with anti-TB therapy may improve outcomes in these critically ill patients.

## 1. Introduction

Intestinal tuberculosis (ITB), a chronic granulomatous disease caused by the Mycobacterium TB complex, is an important form of extrapulmonary tuberculosis (TB). Although TB remains a major global public health concern, intestinal perforation caused by ITB is rare in infancy and poses substantial diagnostic and therapeutic challenges. Its nonspecific initial manifestations may delay diagnosis or lead to misdiagnosis, potentially resulting in poor outcomes. Intestinal perforation as the initial manifestation of ITB in early infancy is exceedingly rare. Several factors may account for the paucity of recognized cases: TB is uncommon in this age group; immature cell-mediated immunity in young infants frequently produces false-negative results on conventional immunological tests; and nonspecific symptoms seldom prompt suspicion of TB before catastrophic complications develop. Consequently, published experience is largely limited to older children and adults. This report addresses the near absence of documented cases and diagnostic and management guidance for primary ITB presenting with multiple intestinal perforations in infants younger than 6 months without known TB exposure. Here, we describe such a case treated at our center.

## 2. Case presentation

A 5-month-and-8-day-old male infant was admitted to our institution on May 14, 2025, with intermittent fever for over 2 weeks and abdominal distension for 1 month, which had worsened during the preceding day. The illness had begun 1 month before admission (Table 1).

**Table 1:** Clinical course timeline of the patient.

Date	Illness d	Event
Mid-April 2025*	D 1	Symptom onset: intermittent fever (up to 39.4°C, predominantly at night); diarrhea developed on d3; oral medication for 5 d with partial relief
Mid-toLate April 2025*		Abdominal distension developed; medical consultation delayed by the parents as general condition appeared acceptable
April 28, 2025*	~D 10	First hospitalization at a local hospital: elevated inflammatory markers (CRP 210.24 mg/L), negative blood cultures; empirical anti-infective therapy with unsatisfactory response
May 4, 2025*	~D 17	Signs of intestinal obstruction; initial exploratory laparotomy at the local hospital: purulent ascites, extensive adhesions, necrotic perforation ~50 cm proximal to the ileocecal junction; ileostomy created; postoperative persistent fever, incisional infection and dehiscence; appendiceal histopathology showed granulomatous nodules with multinucleated giant cells
May 14, 2025	–	After transfer to our hospital, broad-spectrum anti-infective treatment was ineffective.
May 19, 2025	–	Second exploratory laparotomy at our institution: stoma stenosis, loosened suture with re-perforation, segmental bowel stenosis ~15 cm proximal to the stoma; adhesiolysis and double-barrel ileostomy
May 20–21, 2025	–	mNGS of peritoneal drainage fluid (results within 24 h): *Mycobacterium TB* complex 2868,520 reads; primary ITB diagnosed
May 21, 2025	–	Anti-TB therapy initiated: rifampicin + isoniazid + pyrazinamide + ethambutol, combined with linezolid、imipenem and amikacin; parenteral nutrition (90–100 kcal/kg/d), plasma/fibrinogen/albumin support
May 23, 2025	POD 4	Inflammatory markers returned to normal
June 11, 2025	–	Nasogastric feeding established and tolerated (15 mL every 3 h)
June 23, 2025	–	Discharged from PICU; transferred to general surgery ward on June 26 (weight 4.9 kg); continued parenteral nutrition + oral formula (~500 mL/d)
September 6, 2025	–	Discharged home, weight 5.4 kg
October–December 2025	–	Readmissions; ileostomy closure attempted (weight 5.4 kg, length 65 cm); on postoperative d 20, suspected anastomotic leak (incisional inflammation, fecal discharge, abdominal distension) → ileostomy reestablished, During the operation, gray-white nodules were still seen adherent to the mesentery.
January 2026	–	Hospitalized in general surgery: combined parenteral and enteral nutrition; weight 6 kg, length 69 cm
March 2026	–	PICC line–associated infection with sepsis; resolved with treatment; no adverse events attributable to anti-TB therapy
July 2026	~14 mo follow-up	Latest follow-up: weight increased to 9.5 kg; developmental progress encouraging (able to say “mama” and “papa”); ileostomy in situ, reclosure planned after full nutritional recovery; remains on gradual parenteral nutrition weaning

After approximately 10 days of illness, there was an increasing abdominal swelling with irritability, and he was presented to the local hospital. Laboratory tests showed that there were raised inflammatory markers while there was no positive blood culture result. The empirical antimicrobial management was tried but was not successful. After 1 week, the infant developed signs of intestinal obstruction and underwent exploratory laparotomy. The clinical course is summarized in Table 1.

The intraoperative findings revealed that there was abundant purulent discharge and cloudy ascites in the abdominal cavity, with pronounced edema and dilatation of the small bowel. There were extensive adhesions between the intestines and the peritoneum, between intestinal loops, and between the ileocecal area and the sigmoid colon. There was pus, inflammation, and adhesion of the terminal end of the appendix with the sigmoid colon. An area of necrotic bowel with perforation was found about 50 cm proximal to the ileocecal area, with obvious fecal discharge. The postoperative complications include fever, minimal reduction of inflammatory indices, and surgical site infection (Table 1).

Histopathological examination of the appendix revealed acute phlegmonous appendicitis with multinucleated giant cells and granulomatous nodules in the muscular and serosal layers (Fig. 1). The infant was subsequently transferred to our institution for further management.

**Figure 1.**
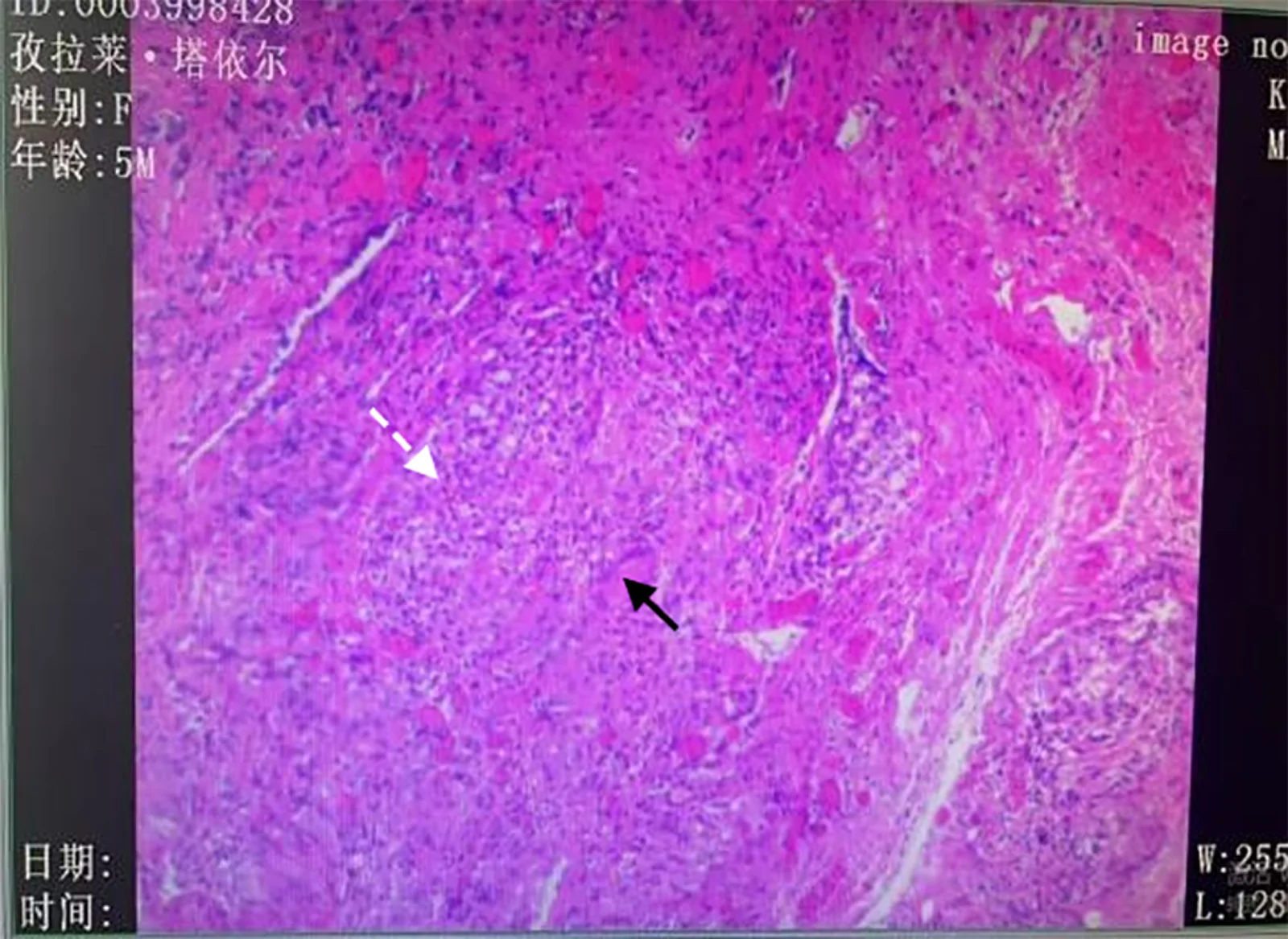
Histopathology of the appendix from the initial surgery (hematoxylin–eosin staining, original magnification × 200), showing multinucleated giant cells (black arrows) and granulomatous nodules (white arrows) in the muscular and serosal layers, on a background of acute phlegmonous appendicitis.

The infant’s birth history was unremarkable. It is noteworthy that the child was capable of lifting the head but did not have the skill of keeping the head steady in a sitting position. At the time of examination, the child had not learned how to roll over and sit supported; he had suffered weight loss as well. Complementary feeding started too early for the child, with introduction of rice cereal at 1 month and meat and noodles at 3 months. Both parents were healthy, and there was no family history of genetic or infectious diseases. On admission, the infant weighed 5 kg, corresponding to a weight-for-age Z-score of <−3 according to the WHO Child Growth Standards and indicating severe malnutrition. His body length was 62 cm, corresponding to a length-for-age Z-score of <−3. He had received bacillus calmette-guérin (BCG) vaccination at birth, and a BCG scar was visible on the left upper arm. HIV serology was negative.

His body temperature was 36.5°C, pulse rate was 166 beats/min, respiratory rate was 38 breaths/minutes, blood pressure was 92/61 mm Hg, and oxygen saturation was 94%. The infant appeared malnourished and was conscious but in poor general condition, with rapid breathing and a dull facial expression. Ecchymoses were present on the left wrist and elbow. His hair was sparse, and his skin was dry with poor turgor. The anterior fontanel was flat and soft. Mild scleral icterus was present, whereas the corneas appeared normal. The pupils were equal and round, with intact light reflexes. The pharynx was hyperemic. Pulmonary auscultation revealed coarse breath sounds bilaterally. Cardiac examination showed a regular rhythm without murmurs. The abdomen was markedly distended, with exaggerated abdominal movement during respiration. A surgical dressing was in place over the lower abdomen. A stoma was present in the left lower quadrant, with a small amount of greenish discharge visible in the stoma bag. Partial wound dehiscence was observed in the right lower quadrant(Fig. 2). Abdominal palpation was limited, and bowel sounds were diminished. Muscle tone was increased, and muscle strength was grade IV. Physiological reflexes were present, pathological reflexes were absent, and meningeal signs were negative.

**Figure 2.**
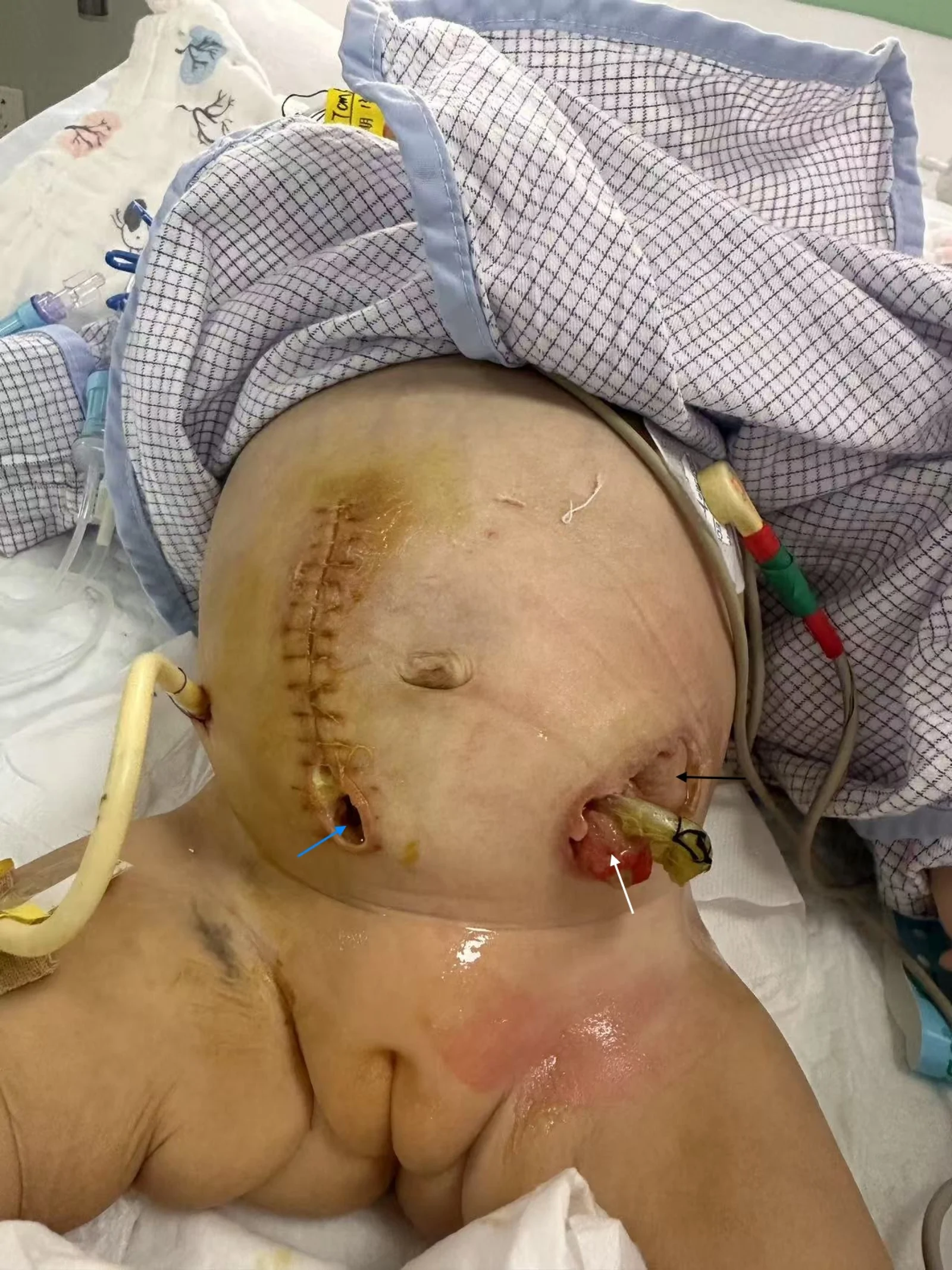
Status of the infant on admission, showing stoma dehiscence, exposed bowel, and right-sided incisional hernia.

### 2.1. Auxiliary examinations

Serial laboratory investigations are summarized in Table 2. At the local hospital, initial laboratory testing showed the following: C-reactive protein (CRP): 210.24 mg/L ↑, Hemoglobin: 79 g/L ↓, Absolute neutrophil count: 11.39 × 10^9^/L ↑, Red blood cells: 4.22 × 10^12^/L, White blood cells: 21.38 × 10^9^/L ↑, Platelets: 449 × 10^9^/L, Serum albumin: 23.1 g/L ↓, Procalcitonin (PCT): 4.25 ng/mL ↑; coagulation function testing on April 30: D-dimer: 5.1 mg/L ↑, Thrombin time: 19.3 seconds, Prothrombin time: 19.4 seconds ↑, Prothrombin activity: 36.70% ↓, International normalized ratio: 1.67 ↑, Activated partial thromboplastin time: 36.80 seconds ↑; Postoperative (status) after exploratory laparotomy: PCT: 0.806 ng/mL ↑; CRP: 85.79 mg/L ↑, Hemoglobin: 89 g/L ↓, Absolute neutrophil count: 13.01 × 10^9^/L ↑, White blood cells: 18.20 × 10^9^/L ↑, Platelets: 217 × 10^9^/L.

**Table 2:** Serial laboratory investigations.

Parameter (reference range)	Outside hospital Apr 28, 2025	Outside hospital, post-op May 12, 2025	On transfer May 14, 2025	May 15, 2025	2nd surgery May 19, 2025	POD 2 May 21, 2025	POD 3 May 22, 2025	POD 4 May 23, 2025
WBC (4.3–14.2 × 10^9^/L)	21.38 ↑	18.20 ↑	20.76 ↑	–	10.11	9.29	6.18	14.96 ↑
ANC (0.6–7.5 × 10^9^/L)	11.39 ↑	13.01 ↑	11.93 ↑	–	5.29	6.17	3.52	8.38 ↑
Hemoglobin (97–183 g/L)	79 ↓	89 ↓	93 ↓	–	69 ↓	88 ↓	88 ↓	86 ↓
Platelets (183–614 × 10^9^/L)	449	217	168 ↓	–	66 ↓	54 ↓	56 ↓	63 ↓
CRP (0–10 mg/L)	210.24 ↑	85.79 ↑	83.32 ↑	–	90.87 ↑	45.33 ↑	15.62 ↑	8.14
PCT (ng/mL)	4.25 ↑	–	–	24.54 ↑*	–	–	–	–
IL-6 (<7.00 pg/mL)	–	–	133.90 ↑*	68.25 ↑	160.85 ↑	–	–	–
Serum albumin (35–50 g/L)	23.1 ↓	–	29.72 ↓	–	–	–	–	–
Total bilirubin (0–21.0 μmol/L)	–	–	59.70 ↑	–	–	–	–	–
PT (11.0–14.3 s)	19.4 ↑	–	22.6 ↑	–	16.9 ↑	–	–	13.5
INR (0.8–1.2)	1.67 ↑	–	2.0 ↑	–	1.5 ↑	–	–	–
Fibrinogen (1.8–3.5 g/L)	–	–	0.77 ↓	–	0.96 ↓	–	–	1.31
aPTT (24–32.8 s)	36.80 ↑	–	47.80 ↑	–	40.90 ↑	–	–	32.2
D-dimer (0–0.55 mg/L)	5.1 ↑	–	2.53 ↑	–	5.61 ↑	–	–	–
AT-III activity (75–125%)	–	–	12.80 ↓	–	13.30 ↓	–	–	–

Upon transfer to our hospital, a complete blood count with reticulocyte count and CRP testing showed the following: CRP: 83.32 mg/L ↑, Hemoglobin: 93 g/L↓, Absolute neutrophil count: 11.93 × 10^9^/L↑, White blood cells: 20.76 × 10^9^/L↑, Platelets: 168 × 10^9^/L, Interleukin-6: 133.90 pg/mL ↑, PCT: 13.87 ng/mL ↑, Serum ferritin: 811.10 ng/mL ↑, Creatinine: 12.93 μmol/L ↓, Total bilirubin: 59.70 μmol/L ↑, Direct bilirubin: 38.58 μmol/L ↑, Total protein: 44.19 g/L ↓, Albumin: 29.72 g/L ↓, Alanine aminotransferase: 7 U/L ↓, bile acids: 82.72 μmol/L ↑, Potassium: 3.56 mmol/L ↓, Serum bicarbonate: 26.10 mmol/L ↑, Calcium: 2.07 mmol/L ↓, Phosphorus: 1.19 mmol/L ↓, Anion gap: 20.46 ↑; Humoral immune function tests: Within normal limits. Coagulation profile (7-item panel): D-dimer: 2.53 mg/L ↑, Fibrin(ogen) degradation products: 7.60 μg/mL ↑, Thrombin time: 25.90 seconds ↑, Prothrombin time: 22.6 seconds ↑, Prothrombin activity: 31.0% ↓, International normalized ratio: 2.0 ↑, Fibrinogen: 0.77 g/L ↓, Activated partial thromboplastin time: 47.80 seconds ↑, Antithrombin III activity: 12.80% ↓; Stool microscopy: White blood cells: 10 to 12/high-power field.

Computed tomography (CT) revealed a disorganized arrangement of the bowel loops, with contiguous segmental dilation, intraluminal gas and fluid accumulation, and air–fluid levels. The bowel loops appeared clustered, with poorly defined interloop spaces, suggesting intestinal obstruction. Scattered punctate foci of gas attenuation were observed beneath the hepatic margin and between some bowel loops, indicating pneumoperitoneum. Increased attenuation was noted in portions of the bowel loops in the right abdomen, and attenuation of the hepatic parenchyma was markedly reduced. Mild dilation of both lateral ventricles and slight widening of the extracerebral spaces in the bilateral frontal and temporoparietal regions were also observed.

Treatment: On admission, the infant received linezolid at 10 mg/kg every 8 hours, imipenem at 25 mg/kg every 6 hours, metronidazole at 7.5 mg/kg every 8 hours, and fluconazole at 12 mg/kg as broad-spectrum anti-infective therapy, together with supportive care. Despite intensive antimicrobial treatment, markers of infection did not improve significantly, while abdominal distension progressed and mental status deteriorated. On May 19, a second exploratory laparotomy was performed and revealed stenosis at the stoma. Intraoperative examination showed severe intestinal congestion, edema, and dilation, with extensive adhesions between the bowel loops. The intestinal wall, mesentery, abdominal wall, and incision site were covered with copious milky-white purulent material. After adhesiolysis, 5 previously repaired incisions were identified in the small intestine. A suture at the distal end of the stoma had loosened, resulting in intestinal perforation. Segmental luminal stenosis was identified approximately 15 cm proximal to the stoma, and a double-barrel stoma was created proximal to the stenotic segment (Figs. 3–4). The original incision was completely closed using tension-reducing sutures. Postoperatively, increased respiratory resistance was observed during mechanical ventilation and was suspected to have resulted from diaphragmatic elevation secondary to increased intra-abdominal pressure. The skin and subcutaneous tissue were closed with 2 interrupted 0 to 0 nylon sutures, whereas the peritoneum and rectus sheath were left unclosed to permit secondary repair if required (Fig. 5). Abdominal drainage fluid was submitted for metagenomic next-generation sequencing (mNGS), which detected Mycobacterium TB complex (2868,520 reads), Acinetobacter baumannii (1622 reads), Enterococcus faecium (719 reads), and human cytomegalovirus (CMV; 8 reads). Nodular tissue specimens collected from 5 sites during the operation were positive for acid-fast bacilli on Ziehl-Neelsen staining. The staining technique done using periodic acid-silver methenamine proved negative for the case, ruling out the possibility of a fungal cause of infection. It is important to note that there was no mycobacterial culture and GeneXpert/MTB-PCR done; these are limitations in the diagnostic process. The patient’s diagnosis was ITB, which was proven through the absence of any other manifestation of the disease in other body parts, normal results on the cranial and thoracic CT scan, and negative family history of TB. There was a delay of about 1 month before the diagnosis of a life-threatening condition was made. This delay may have reflected a combination of possible socioeconomic barriers to healthcare access, nonspecific clinical manifestations in infants, inadequate recognition of pathologic hallmarks, and delayed use of mNGS. The anti-infective regimen was subsequently modified to include linezolid at 10 mg/kg every 8 hours, imipenem at 25 mg/kg every 6 hours, amikacin at 7.5 mg/kg every 12 hours, rifampin at 8 mg/kg/day, isoniazid at 10 mg/kg/day, pyrazinamide at 35 mg/kg/day, and ethambutol at 20 mg/kg/day.

**Figure 3.**
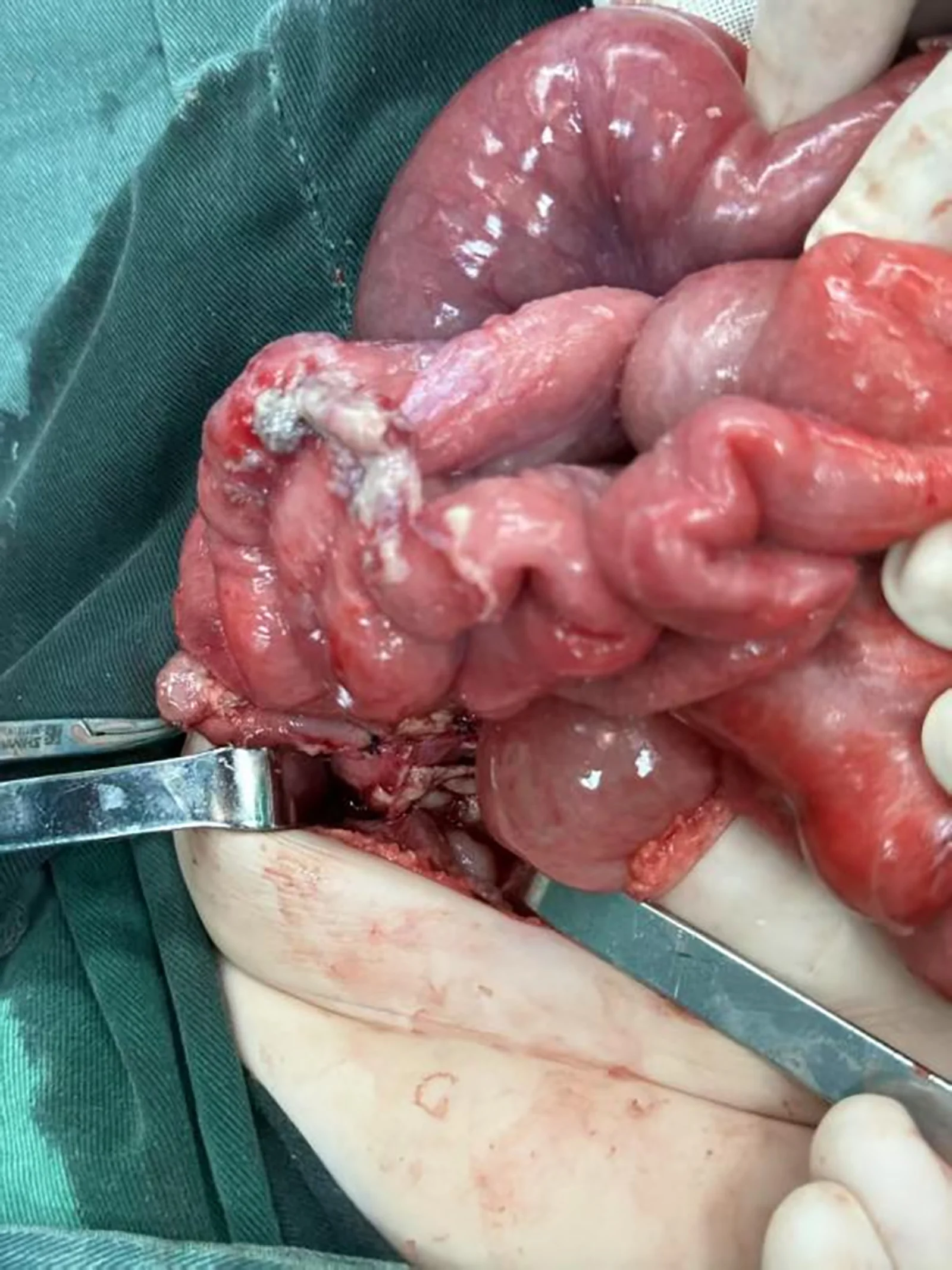
Intraoperative view showing severe bowel wall edema with multiple whitish nodules adhered to the intestinal serosa.

**Figure 4.**
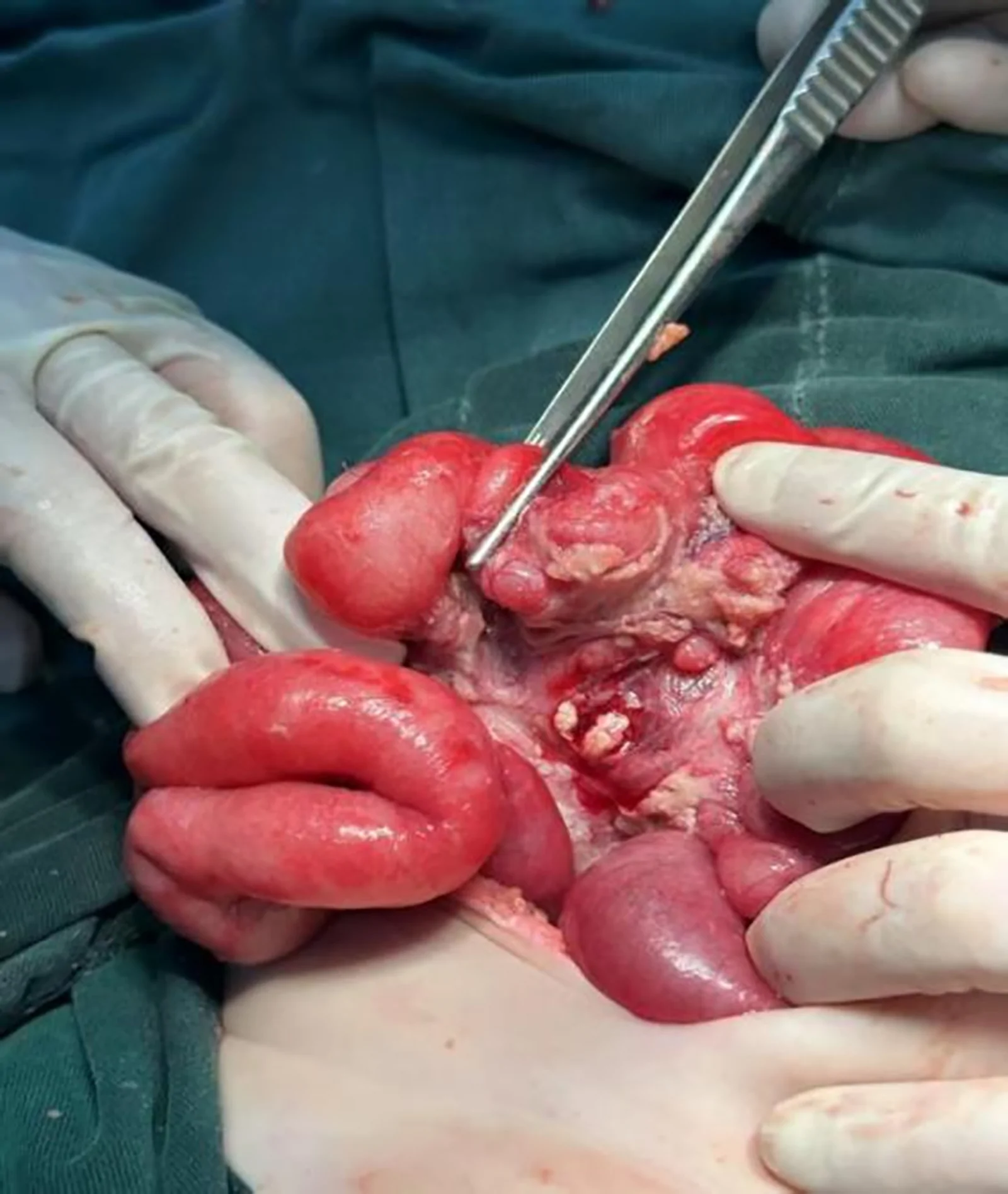
Intraoperative close-up view showing the serosal whitish nodules in detail.

**Figure 5.**
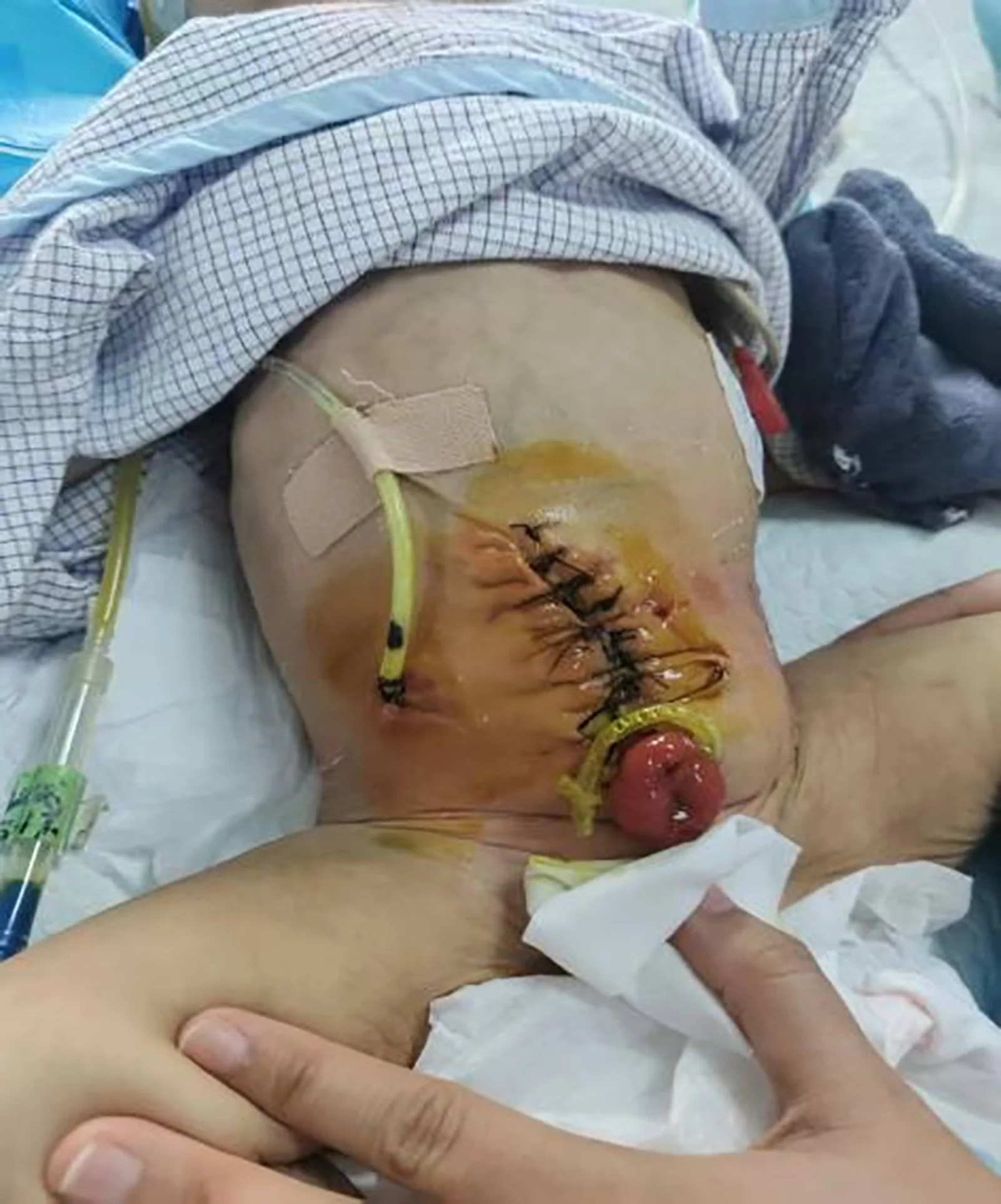
Post-reoperation status showing recreation of ileostomy with viscous white purulent discharge from a drainage tube after secondary laparotomy in our institution. PPD = purified protein derivative.

Nutritional rehabilitation and supportive care. Parenteral nutrition was initiated immediately after admission, providing 90 to 100 kcal/kg/day. Albumin was administered intermittently at 6 g per infusion whenever the serum albumin concentration fell below 30 g/L, with 7 infusions administered in total. Enteral feeding with infant formula was intermittently attempted through a nasogastric tube. Abdominal distension and vomiting were managed with phentolamine and a traditional Chinese herbal preparation intended to promote intestinal motility, together with oral probiotics. Beginning on June 11, nasogastric feeding was successfully established at 15 mL every 3 hours (24 kcal/kg/day) and was well tolerated, allowing the gradual advancement of enteral nutrition.

Postoperative intensive care. The infant was successfully weaned from mechanical ventilation after the second laparotomy. Coagulopathy was corrected with 7 plasma transfusions (50 mL each) administered between May 15 and May 20, together with a single infusion of 0.25 g of fibrinogen. Elevated liver enzyme levels were managed with compound glycyrrhizin. Intravenous immunoglobulin was administered beginning on June 13 at 2.5 g per dose for a total of 3 doses as immunological support because of the infant’s young age, severe infection, and malnutrition.

Wound and stoma management. The patient was fitted with a drainage bag and changed whenever necessary. The peristomal area was cleaned every day using normal saline solution, while peristomal irritant dermatitis was addressed using epidermal growth factor. The surgical wound, including the dehisced portion of the incision, was dressed daily. The nasogastric tube was removed on June 22, and the infant was transferred out of the ICU on June 23, 2025, in stable condition with established enteral feeding.

Treatment outcome: The patient had shown signs of improvement as indicated by the lack of recurrence of postoperative obstruction, good weight gain, and an increased level of mental alertness. The infection indices came to normal on postoperative day 4 after the second surgery. Most ITB patients show good prognosis if they are managed through a multi-modal treatment method that combines surgery and antituberculosis medication. The infant responded well to the treatment protocol without any side effects from the medication.

Follow-up. At the time of reporting, the infant had been followed for more than 12 months after the second laparotomy (May 2025 to July 2026). Discharge from the pediatric intensive care unit (ICU) took place on June 23, 2025, while the transfer to the general surgical ward happened on June 26, 2025, weighing 4.9 kg. Parenteral nutrition was maintained in combination with formula feeding at an approximate intake of 500 mL/day (90–100 kcal/kg/day). He was discharged home on September 6, 2025, weighing 5.4 kg.

Ileostomy closure was attempted during readmissions in October and December 2025, while the baby weighed 5.4 kg and measured 65 cm in height. There were gray-white nodules found in the mesentery during the time of surgery. Nevertheless, the acid-fast stain for the said nodules was negative which is different from the positive stain noted in the same nodules obtained in May 2025 before anti-TB treatment. On postoperative day 20, the patient showed signs of erythematous and edematous incisions, fecal leak, and marked abdominal distention raising concerns about an anastomotic leak. Thus, the ileostomy was reinserted. As of January 2026, the patient is under hospitalization and receiving both intravenous and oral nutritional regimens. The patient’s weight was recorded as 6 kg and height of 69 cm at this point. The patient experienced an infection related to the insertion of peripherally inserted central catheter (PICC) in March 2026, together with sepsis, which was reversed upon management. There have been no cases of any side effects from the TB treatment such as liver toxicity, hemotoxicity, and others. In the latest follow-up done in July 2026, the weight of the patient had increased to 9.5 kg, while the development is positive with the ability of the patient to vocalize “mama” and “papa.” The ileostomy remains in situ, with repeat closure planned after complete nutritional recovery.

A literature search of PubMed and Google Scholar was conducted in August 2025 using the terms (“infant” [MeSH]) AND (“TB, gastrointestinal” [MeSH] OR “ITB”) AND (“intestinal perforation” [MeSH]), without language restrictions. Reports describing ITB complicated by intestinal perforation in children were screened, and cases involving infants were identified for comparison (Table 3).^[1–9]^

**Table 3:** Comparison of previously reported pediatric cases of ITB complicated by intestinal perforation.

Author, yr (Ref.)	Age/Sex	TB exposure history	Diagnostic method	Perforation site/number	Treatment	Outcome
Dhar et al 1990^[1]^	7 mo–>10 yr (youngest 7 mo; 53% >10 yr); sex NR (case series, n = 19)	NR	Postoperative histopathology (preoperative diagnosis often difficult)	Terminal ileum 63.2%, jejunum 26.3%; multiple perforations 15.8%	Surgery (for acute peritonitis)	Mortality 63% (12/19); preoperative condition was the main prognostic factor
Agrawal et al 1995^[2]^	Premature neonate (congenital TB); sex NR	Congenital (maternal transmission, miliary TB)	Clinical + pathology (congenital miliary TB)	Multiple intestinal perforations	Anti-TB chemotherapy + surgical repair	Survived
Mahajan et al 2007^[3]^	6 mo/Male	NR	Histopathology (caseating epithelioid granulomas in ileal mucosa/submucosa)	Ileocecal region (intussusception with localized bowel necrosis); single segment	Segmental bowel resection + end-to-end anastomosis	Survived
Acer et al 2008^[4]^	2 yr/Female	Congenital miliary pulmonary TB	Histopathology (granulomas on appendiceal biopsy); characteristic pale yellow serosal nodules intraoperatively	Multiple jejunoileal perforations	Surgery (for perforation after sepsis)+anti-TB therapy	Improved (recovered after surgery and anti-TB therapy)
Mirza et al 2011^[5]^	Mean 8.3 yr; sex NR (case series, n = 5 with perforation among 18 surgical ITB cases)	NR	NR (surgical series)	NR	Surgery	NR
Singal et al 2012^[6]^	7 yr/Female	NR	Intraoperative findings (multiple white nodules on bowel wall) + pathology	Solitary ileal perforation	Surgical intervention + anti-TB therapy	Clinical improvement
Nahida et al 2018^[7]^	10 yr/Male	NR	Histopathology	Sealed-off perforation between colon and small bowel, with mesenteric mass	Surgery (initially managed as acute appendicitis) + anti-TB therapy	NR (diagnosis confirmed postoperatively)
Lal et al 2020^[8]^	<12 yr; sex NR (case series, n = 6 with perforation among 218 abdominal TB cases)	NR	NR	NR	NR	NR
Liu et al 2023^[9]^	18 mo/Male	Positive family history of TB	Pathology/clinical	Intestinal perforation complicated by umbilical enterocutaneous fistula and ileovesical fistula	Surgery + anti-TB therapy	Stable (generally stable condition, moderate inflammatory markers)
Present case, 2026	5 mo/Male	None identified	mNGS of peritoneal drainage fluid (MTBC 2868,520 reads) + histopathology (granulomatous inflammation with multinucleated giant cells)	Multiple perforations: distal ileum (~50 cm proximal to the ileocecal junction), appendiceal region, and re-perforation at the stoma	Two laparotomies (initial surgery + adhesiolysis and double-barrel ileostomy) + four-drug anti-TB therapy (HRZE) combined with linezolid and amikacin	Improved; ileostomy in situ after failed closure (suspected anastomotic leak), satisfactory weight gain (9.5 kg at 14-mo follow-up)

This study was approved by the Ethics Committee of the Children’s Hospital of Xinjiang Uygur Autonomous Region. Written informed consent was obtained from the infant’s parents for publication of this case report and the accompanying images and clinical data.

## 3. Discussion

According to the WHO Global TB Report 2024, approximately 10 million people develop TB annually, of whom approximately 12% are children aged 0 to 14 years. In China, however, children account for only approximately 1% of reported cases, which may contribute to low clinical suspicion of TB in this age group.^[10]^ We describe an infant with ITB whose initial manifestation was intestinal perforation, an exceedingly uncommon presentation in infancy that is readily misdiagnosed.

Proposed routes of gastrointestinal infection with Mycobacterium TB include direct seeding after the ingestion of infected sputum, hematogenous dissemination, and the consumption of contaminated food, particularly in developing countries.^[11]^ Host genetic factors may also confer susceptibility to TB. Several genetic variants have been reported more frequently in patients with TB, and inherited susceptibility in children may manifest as Mendelian susceptibility to mycobacterial disease (MSMD).^[12]^ In the present case, the absence of a relevant family history or evidence of hematogenous dissemination was considered consistent with primary ITB. We speculate that contaminated food may have been the source of infection, although this route could not be confirmed. The BCG vaccine provides protection across age groups. Although this infant had an unremarkable birth history and received BCG vaccination at birth, he nevertheless developed TB. A report in The Lancet Global Health indicated that pulmonary TB predominantly results from primary infection, whereas extrapulmonary TB is more often associated with hematogenous dissemination or reactivation of latent infection. BCG may therefore be more effective in preventing pulmonary lesions following primary infection but provide only limited protection against extrapulmonary TB.^[13]^

The presentation of ITB tends to be unusual and vague,^[14]^ hence creating specific problems with the diagnosis of this condition in infant cases. In the present case, the infant had irregular fever, abdominal distention, and good mental status. The manifestation may have been underestimated by the parents resulting in delayed medical treatment. Obstruction, perforation, and intra-abdominal infection are complications of ITB. Nonetheless, it is not always easy to differentiate ITB in infants who do not present with any classical signs of TB infection, which include exposure to TB, positive purified protein derivative (PPD) tuberculin test, or a specific T-cell immune response to TB or X-ray evidence of pulmonary lesions. Other conditions of an acute abdomen that can mimic ITB are necrotizing enterocolitis and appendicitis, or inflammatory bowel disease, particularly Crohn’s disease (CD).^[15]^

Nahida et al documented a case report of an occluded coloenteric fistula in a child aged 10 years which presented like acute appendicitis and confirmed by histopathological analysis.^[7]^ The cases of a 6-month-old baby with ileocecal intussusception due to caseating granulomatous tubercular ileitis have been described by Mahajan et al^[3]^ All these cases highlight the fact that ITB can easily be misdiagnosed as other causes of acute surgical abdomen.

Diagnosis of the case was very challenging. It was revealed through laparotomy that there were intestinal adhesions, perforations with necrosis, abscesses in the abdomen, and granulomatous appendicitis. These features suggested infantile CD. Despite the fact that infantile CD is very rare, granulomatous inflammation in the appendix is relatively characteristic, as well as intestinal perforations. In addition, the poor reaction to powerful broad-spectrum antibiotics raised the questions concerning the diagnosis. Infantile CD often shows suboptimal reaction to steroids, and specific treatment can be necessary.

However, the extensive differential diagnosis of granulomatous inflammation of the intestine also included other infectious causes and immunologic disorders. Even though CD was suspected in the patient, her age, the mNGS data, and the anti-TB treatment response excluded this diagnosis. The possibility of fungal infection was excluded due to the absence of any fungal sequences in the broad and unbiased pathogen detection performed by mNGS, the absence of fungal elements in periodic acid-silver methenamine staining of nodular specimens, and their absence in the histopathologic evaluation. Furthermore, the presence of Yersinia infection causing granulomatous ileitis was also not confirmed using mNGS. Primary immunodeficiency disorders such as MSMD and chronic granulomatous disease may predispose infants to severe mycobacterial disease. In the present case, the humoral immunity was normal, and the history revealed no cases of recurrent or unusual infections in the family. However, cellular immune function and genetic analysis have not been performed in the patient. Therefore, the granulomatous inflammation, positivity of acid-fast staining, numerous reads of M. TB on mNGS, and anti-TB treatment response are more compatible with ITB.

Conventional diagnostic tests for TB, including the PPD skin test, and the interferon-gamma release assay, were all negative in this infant (Fig. 6). Due to the severity of his condition, an endoscopic biopsy was not feasible in order to perform a tissue biopsy. Such a difficulty is mainly attributed to the low development of the cell-mediated immune system and the poorly developed Th1 response of infants. It has been shown that immunological tests yield false negatives in up to 65% of TB patients aged below 12 months old. The above-mentioned sequence is different from what is seen in adults and indicates that the sole use of immunological tests might be 1 of the reasons for delayed diagnosis. According to Chow et al (2002), mortality among those suffering from tuberculous peritonitis was as high as 51% due to the delay in treatment while waiting for culture results of ascites.^[16]^ Wang et al (1998) reported a mortality rate as high as 31%.^[17]^ Katigbak et al (2005) described a 15-month-old infant in whom delayed diagnosis and treatment while awaiting pathologic and culture results led to end-stage circulatory failure.^[18]^ These reports underscore the severe adverse effects of delayed diagnosis and treatment on prognosis.

**Figure 6.**
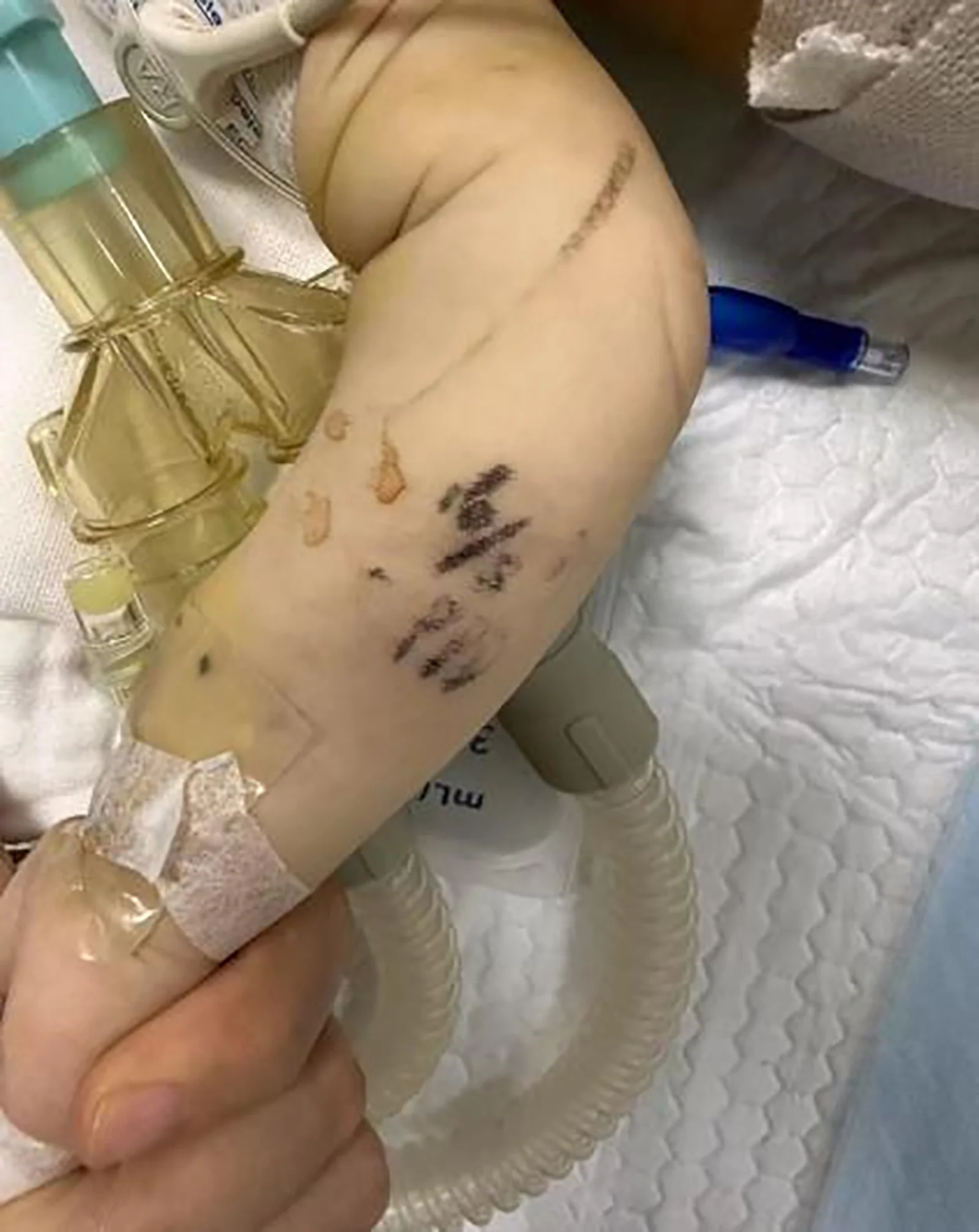
Negative tuberculin skin test (PPD) result in the infant. PPD = purified protein derivative.

The role of mNGS, which makes possible rapid, comprehensive, and extremely sensitive detection of pathogens from clinical specimens, was critical to making this diagnosis. The mNGS test performed on postoperative samples of abdominal drainage fluid provided results in <24 hours, demonstrating 2868,520 reads corresponding to the Mycobacterium TB complex. The main strengths of mNGS include rapidity (≤24 hours), sensitivity, especially with regards to low abundance and difficult-to-cultivate pathogens, and comprehensive detection of many pathogens at once. In critically ill patients with diagnostically challenging presentations or atypical immune responses, mNGS can serve as a valuable adjunctive tool, enabling timely pathogen identification and earlier initiation of targeted treatment.

Several converging lines of evidence supported the Mycobacterium TB complex (MTBC) as the primary pathogen rather than merely 1 component of a polymicrobial postoperative infection. First, MTBC is not a colonizing organism. Unlike Acinetobacter baumannii and Enterococcus faecium, which are commonly associated with hospital environments and enteric flora, respectively, MTBC is not part of the commensal microbiota. Its detection at a normally sterile site with an extremely high read count (2868,520 reads), several orders of magnitude higher than those of the other organisms, cannot reasonably be attributed to colonization or contamination. Second, in an infant with multiple intestinal perforations and fecal contamination of the peritoneal cavity, low-level translocation of enteric and nosocomial organisms would be expected. Their detection was therefore more consistent with secondary translocation than with a primary etiologic role. Third, the clinical course was inconsistent with a primary acute bacterial infection. Fever and abdominal distension began 1 month before admission and preceded any surgical intervention or nosocomial exposure, indicating an indolent, subacute course characteristic of TB. Moreover, the infant continued to deteriorate despite broad-spectrum antimicrobial therapy covering A. baumannii and E. faecium with imipenem, linezolid, and metronidazole. Inflammatory markers did not normalize until anti-TB therapy was added, and repeated blood cultures remained negative. Fourth, granulomatous inflammation with multinucleated giant cells on histopathologic examination and positive acid-fast staining of the mesenteric nodules provided independent morphologic evidence of TB. Fifth, marked clinical improvement occurred only after anti-TB treatment was initiated. Accordingly, A. baumannii and E. faecium were considered secondary nosocomial or translocated organisms and were covered by the broad-spectrum antibacterial regimen. The 8 CMV reads were interpreted as evidence of low-level reactivation during critical illness, and ganciclovir was administered.

Previous data indicate that approximately 14% of TB cases among children younger than 15 years are diagnosed as extrapulmonary TB. The proportion of extrapulmonary TB is significantly higher among children younger than 5 years than among older age groups.^[19]^ Intestinal perforation is a rare complication of ITB, with reported incidence rates of 0% to 16.8% in adults and 3% to 4% in children.^[20–22]^ Although any intestinal segment may be involved, the distal ileum is most commonly affected. In approximately 90% of adult cases of tuberculous intestinal perforation, the perforation is solitary. Although cases in adults are reported more frequently, multiple perforations remain uncommon.^[23,24]^ The patient in the present case was only 5 months old, had multiple intestinal perforations, and lacked a clear history of TB exposure, substantially increasing the diagnostic difficulty.

The pathophysiology of intestinal perforation seen in ITB has mainly been described from data obtained in adults.^[25,26]^ After the initial infection and contact between the bacilli and lymphoid tissue-filled terminal ileum, the resulting inflammatory process of caseation may progress to form ulcers through the wall of the intestines. Mesenteric endarteritis may also contribute to ischemic necrosis of the bowel wall.^[27,28]^ People who suffer from malnutrition and poor cell-mediated immunity, such as the baby in question, are at risk of developing transmural perforations from microperforations.^[29,30]^

The immune response in infants to Mycobacterium TB is immature, which results in high false negatives in immunologic testing for this condition. TB in this age group tends to be insidious in nature but has a quick progression in many cases. Malnutrition, as seen in this case, is another factor that can affect immunity and create a vicious cycle. Premature feeding may have led to malnutrition and bowel flora imbalance in this patient, although there is no way to link the 2 factors directly to TB. Developmental delay seen in the patient could be due to the illness and not any specific neurologic problem. On admission, the patient had critical illness due to intestinal perforation, intra-abdominal infection, intestinal adhesions, stomal stricture, incisional hernia, wound dehiscence with infection, and high levels of inflammation. This severe clinical instability at times precluded timely reoperation.

Following the acquisition of the mNGS analysis results, the management of the patient corresponded to the traditional anti-TB combination of 4 drugs. In order to ensure synergistic mycobacterial effects and coverage for Gram-negatives, imipenem, linezolid, and amikacin were retained, while ganciclovir was used for CMV reactivation treatment. The infection control, coagulation abnormalities correction, nutritional support, and proper care for the wound/stoma were important for the patient’s recovery process especially taking into account an immature abdominal wall and low tissue healing ability.

Literature Review: PubMed and Google Scholar were searched in August 2025 using the terms (“infant”[MeSH]) AND (“TB, gastrointestinal”[MeSH] OR “ITB”) AND (“intestinal perforation”[MeSH]), with no language restrictions. Reports describing ITB complicated by intestinal perforation in children were screened. ITB constitutes a rare form of pediatric TB and an even rarer cause of intestinal perforation in infants. The case of congenital intestinal TB with intestinal perforation reported by Agrawal and Rehman^[2]^ needs to be distinguished from our case. In their case report, a preterm infant with congenital miliary TB was documented to have had multiple intestinal perforations, a situation entirely different from our current case in terms of the mode of acquisition (vertical congenital vs postnatal), maturity of the infant, and the entire clinical picture. Among postnatally acquired cases with a known age, the youngest previously reported patients were a 6-month-old infant^[3]^ and a 7-month-old infant.^[1]^ Based on our literature search, the present case is, to our knowledge, among the youngest reported cases of postnatally acquired primary ITB presenting with multiple intestinal perforations in an infant without a known history of TB exposure. Previously reported cases are summarized in Table 3.

Historical evidence from the 1930 Lübeck incident supports the plausibility of a primary enteral route of infection. In that incident, infants who were orally administered BCG contaminated with virulent Mycobacterium TB developed predominantly intestinal disease, including gastrointestinal complications such as perforation.^[31]^

R. L. Agrawal reported congenital TB in a premature infant who developed multiple intestinal perforations. This presentation was likely associated with the infant’s severely impaired immunity, high bacillary burden, and the virulence of the Mycobacterium TB. Management required combined anti-TB pharmacotherapy and surgical repair.^[2]^

The principal characteristics of these reports are summarized in Table 3; only the main comparative findings are discussed here. Dhar Ajay noted that 123 cases of intestinal perforation in children had been treated surgically at Kalawati Saran Children’s Hospital from 1978 to 1988, of which 19 had tuberculous enteritis. The age range included 7 months-old child and about 53% (10/19) of the patients had more than 10 years.^[1]^ No specific details were mentioned for the case of 7 months-old baby. Of the 19 patients of tuberculous intestinal perforation, there was a mortality rate of 63% (12/19). Preoperative clinical condition was considered as the most important prognostic factor. Most frequent site of tuberculous intestinal perforation was seen to be in terminal ileum (63.2%) and jejunum (26.3%). Multiple perforation was present in about 15.8% patients. Acute peritonitis was a common presentation, but preoperative diagnosis was often difficult, with some cases diagnosed definitively only by postoperative histopathologic examination. Malnutrition, toxemia, and fecal peritonitis were associated with poor outcomes.

An 18-month-old boy suffering from abdominal pain was diagnosed with ITB with perforation resulting in secondary umbilical enterocutaneous fistula and ileovesical fistula according to Liu et al The patient had a family history of TB, increased inflammatory indexes, and stable state of health.^[9]^ A 2-year-old girl suffering from congenital miliary TB of the lungs and developing sepsis and then intestinal perforation was presented in the work of Acer et al^[4]^ The presence of small pale yellow nodules on the surface and in the layers of the intestine is considered to be specific signs which may indicate the development of TB during the course of the laparotomy or laparoscopy. In the current case, inappropriate infection control and severe distention of the abdomen required the assessment of risks for performing the surgery and conservative therapy.

Singal reported a 7-year-old girl with abdominal TB who presented primarily with peritonitis and intestinal obstruction accompanied by a solitary ileal perforation. Intraoperative examination revealed multiple white nodules on the intestinal wall. The patient improved clinically after surgical intervention and anti-TB therapy.^[6]^

This case emphasizes that, in TB-endemic regions, ITB should be strongly considered in infants without a known history of TB exposure who present with unexplained prolonged fever, abdominal distension, malnutrition, and developmental regression, particularly when accompanied by intestinal obstruction, perforation, an intra-abdominal abscess, and granulomatous histopathologic changes. Conventional TB tests may be negative. In critically ill patients with complex suspected infections, especially infants and young children who may have atypical immune responses, mNGS may facilitate timely pathogen identification when conventional investigations are inconclusive, together with careful assessment and management of nutritional status. In our patient, acid-fast staining of grossly similar mesenteric nodules changed from positive in May 2025, before treatment, to negative in October 2025, after 5 months of anti-TB therapy. Although this conversion does not provide definitive evidence because of the limited sensitivity of acid-fast staining and the possibility of sampling variation, it is consistent with effective bacillary clearance. Together with normalization of inflammatory markers and sustained weight gain, this finding supports an adequate treatment response. The diagnosis of primary ITB was based primarily on unremarkable cranial and thoracic CT findings and the absence of known TB exposure. Although this interpretation is reasonable, occult pulmonary disease and unidentified exposure cannot be completely excluded.

## 4. Conclusion

We report a rare case of primary ITB presenting with multiple intestinal perforations in a 5-month-old infant. This case emphasizes that, in TB-endemic regions, ITB should be considered in infants with unexplained prolonged fever, abdominal distension, and signs of intestinal obstruction or perforation, even when conventional TB investigations are negative. When conventional investigations are inconclusive, mNGS may facilitate early diagnosis. Early recognition, timely anti-TB therapy combined with appropriate surgical intervention, and meticulous nutritional and supportive care are essential for improving outcomes in critically ill infants.

## Author contributions

**Conceptualization:** Bei Wang, Yipaguli Simijiang, Yanfei Cui.

**Data curation:** Bei Wang, Yipaguli Simijiang, Yusufu Akemu, Nuerai Shawutali.

**Formal analysis:** Bei Wang, Yipaguli Simijiang, Gulibiya Kaheman.

**Supervision:** Yanfei Cui.

**Validation:** Yanfei Cui.

**Writing – original draft:** Bei Wang, Yipaguli Simijiang, Gulibiya Kaheman.

**Writing – review & editing:** Bei Wang, Yipaguli Simijiang, Yanfei Cui.
